# POCUS Examination of the Mediastinum in Children: A Simplified and Standardized Protocol for Pulmonary Tuberculosis

**DOI:** 10.24908/pocusj.v10i02.19176

**Published:** 2025-11-17

**Authors:** Isabelle Munyangaju, Lucia Carratala-Castro, Sozinho Acacio, José Miguel Escudero Fernández, Antoni Soriano-Arandes, Maria Espiau, Begoña Santiago Garcia, Alicia Hernanz-Lobo, Ángel M. Lancharro Zapata, Aleix Soler-Garcia, Enrique Ladera, Antoni Noguera-Julian, Angela Manzanares, Daniel Blazquez, Elisa Aguirre Pascual, José Massingue, Jessica Dalsuco, Justina Bramugy, Isabelle Thierry-Chef, Quique Bassat, Danilo Buonsenso, Elisa Lopez-Varela, Xavier Serres-Créixams

**Affiliations:** 1 Barcelona Institute for Global Health - Hospital Clínic, Universitat de Barcelona; 2 Facultat de Medicina i Ciències de la Salut, Universitat de Barcelona (UB), Barcelona, Spain; 3 CIBER Epidemiología y Salud Pública (CIBERESP), Av. Monforte de Lemos, 3-5. Pabellón 11. Planta 0 28029 Madrid, Spain; 4 Centro de Investigação em Saúde de Manhiça (CISM), Manhiça, Mozambique; 5 Instituto Nacional de Saúde (INS), Ministério da saúde-Moçambique; 6 Radiologia Pediàtrica, Hospital Universitari Vall d'Hebron, Barcelona, Spain; 7 Unitat de Patologia Infecciosa i Immunodeficiències de Pediatria (UPIIP), Hospital Universitari Vall d'Hebron, Barcelona, Spain; 8 Vall d'Hebron Research Institute, Infection and Immunity in Children, Barcelona, Spain; 9 Pediatric Infectious Diseases Department, Gregorio Marañón University Hospital; Gregorio Marañón Research Health Institute (IiSGM), Madrid, Spain; 10 Centro de Investigación Biomédica en Red de Enfermedades Infecciosas (CIBERINFEC), Instituto de Salud Carlos III, Madrid, Spain; 11 Translational Research Network in Pediatric Infectious Diseases (RITIP). Madrid, Spain; 12 Radiologia Pediàtrica, Hospital General Universitario Gregorio Marañón, Madrid, Spain; 13 Malalties Infeccioses i Resposta Inflamatòria Sistèmica en Pediatria, Servei de Malalties Infeccioses i Patologia Importada, Institut de Recerca Pediàtrica Sant Joan de Déu, 08950 Barcelona, Spain; 14 Radiologia Pediàtrica, Hospital Sant Joan de Déu, Barcelona, Spain; 15 Centro de Investigación Biomédica en Red de Epidemiología y Salud Pública (CIBERESP), 28029 Madrid, Spain; 16 Departament de Cirurgia i Especialitats Medicoquirúrgiques, Facultat de Medicina i Ciències de la Salut, Universitat de Barcelona, 08036 Barcelona, Spain; 17 Red de Investigación Translacional en Infectología Pediátrica RITIP, 28029 Madrid, Spain; 18 Pediatric Infectious Diseases Unit. Hospital Universitario 12 de Octubre. Instituto de Investigación Hospital 12 de Octubre. Universidad Complutense, Madrid, Spain; 19 Radiologia Pediàtrica, Hospital Universitario 12 de Octubre, Madrid, Spain; 20 Hospital Distrital de Manhiça, Ministerio de Saúde, Mozambique; 21 Universitat Pompeu Fabra (UPF), Barcelona, Spain; 22 ICREA, Pg. Lluís Companys 23, 08010 Barcelona, Spain; 23 Departament de Pediatria, Hospital Sant Joan de Déu, Universitat de Barcelona, Barcelona, Spain; 24 Department of Woman and Child Health and Public Health, Fondazione Policlinico Universitario A. Gemelli IRCCS, Rome, Italy; 25 Area Pediatrica, Dipartimento di Scienze della Vita a Sanità Pubblica, Università Cattolica del Sacro Cuore, Rome, Italy; 26 Global Health Research Institute, Istituto di Igiene, Università Cattolica del Sacro Cuore, Roma, Italy; 27 Radiodiagnóstico, Ecografia intervencionista, Hospital Universitario Vall d'Hebron, Barcelona, Spain

**Keywords:** Ultrasonography, paediatric POCUS, Mediastinum, Ultrasound, Lymphadenopathy, Children, Paediatric tuberculosis

## Abstract

**Background:**

Point of care ultrasound (POCUS) is increasingly recognized as a valuable tool for mediastinal assessment in children, particularly in resource-limited settings where advanced radiological options such as computed tomography (CT) scans are often unavailable. In high-income countries, POCUS is gaining traction as a complementary imaging method, offering a safer, radiation-free alternative.

**Methods:**

To overcome the operator-dependent nature of mediastinal POCUS, a standardized protocol was developed. The protocol includes clear techniques and detailed descriptions of normal and pathological findings, aiming to enhance consistency and diagnostic accuracy.

**Results:**

The standardized protocol improved reliability in mediastinal POCUS assessments, especially in the context of paediatric pulmonary tuberculosis, a condition often marked by lymph node involvement. Given the challenges of obtaining respiratory samples in children and their typically low diagnostic yield, POCUS emerged as a particularly suitable diagnostic modality.

**Conclusions:**

Mediastinal POCUS, guided by a standardized protocol, represents a safe, affordable, point-of-care, and non-ionizing option for identifying mediastinal lymphadenopathy. Its application holds promise for improving the diagnosis of paediatric tuberculosis, especially in settings with limited access to advanced radiological imaging.

## Introduction

Tuberculosis (TB) remains a significant global health challenge, particularly in paediatric populations. Children below 5 years of age are more susceptible to developing severe and disseminated forms of TB, which can lead to substantial morbidity and mortality if not diagnosed and treated promptly [1–3]. Of the 10.6 million new TB cases globally in 2022, 12% were children, and of the estimated 1.3 million TB deaths, 16% were children [1]. Despite the high burden of paediatric TB, diagnosing the disease in children is particularly challenging. Young children often present with non-specific symptoms, and the paucibacillary nature of the disease in this age group results in low sensitivity of conventional microbiological diagnostic tests in difficult-to-obtain respiratory samples, such as induced sputum and gastric aspirates. Consequently, many cases of paediatric TB are either missed or diagnosed late, contributing to delays in treatment initiation and risk on adverse health outcomes [4–7].

The diagnostic challenges of paediatric TB are also compounded by limitations in the most commonly used radiological diagnostic techniques [8,9]. Chest X-rays (CXRs) are an important tool in TB diagnosis, recommended by the World Health Organization guidelines for diagnosing TB in children and for TB contact tracing [10]. In paediatric TB, mediastinal lymph node involvement, often characterized by significant lymph node enlargement, is the most frequent anatomical manifestation, more prevalent than pulmonary consolidation or pleural effusion [11]. However, CXR has its limitations, including inter-observer variability and the inability to differentiate between active TB and other respiratory conditions [12–16]. In diagnosing paediatric TB, the detection of specific pathological features on CXRs—such as lymphadenopathy, pleural effusions, or cavities—has only 50% sensitivity and 90% specificity when compared to confirmed TB cases, based on Graham's clinical classification [17]. Computed tomography (CT) scans offer greater diagnostic accuracy in paediatric TB by providing detailed imaging of lung structures, enabling the detection of subtle or complex disease features that may be missed on CXRs [18,19]. Imaging plays a crucial role in the diagnosis and management of TB, providing valuable information that complements clinical and microbiological data in diagnostic algorithms [7,16]. However, the prohibitive costs of installing and maintaining X-ray and CT scan services, the need for sophisticated infrastructures, and the stringent radiation protection standards required, make these methods less accessible in resource-limited settings [20–25].

In contrast, point of care ultrasound (POCUS) offers several advantages over traditional radiological methods, particularly for paediatric TB diagnosis. POCUS is a clinician-performed, non-invasive, radiation-free imaging modality that is being increasingly utilized for the diagnosis of various medical conditions, including TB [26–28]. Similar to the emerging trends in the diagnosis of paediatric pneumonia, several studies have shown the potential role of POCUS in diagnosing paediatric TB [26–34]. The use of POCUS for mediastinal imaging in paediatric TB can provide real-time dynamic assessments of the mediastinum, allowing for the visualization of lymphadenopathy and other pathological changes associated with TB [12,35–40].

The advantages of POCUS extend beyond its diagnostic capabilities. Ultrasound machines are generally more affordable and portable compared to X-ray and CT scan equipment, making them ideal for use in low- and middle-income countries where healthcare resources are limited [16,20,41–43]. Additionally, ultrasound does not involve ionizing radiation, thereby eliminating the risks associated with radiation exposure, or the need of intravenous contrast. This is particularly important in paediatric populations, where minimizing radiation exposure is crucial to reduce the risk of long-term adverse effects [22].

While traditional imaging techniques such as CXR and CT scans remain integral to the diagnostic algorithms for paediatric TB, their limitations necessitate the exploration of alternative methods, particularly when targeting patients in low- and middle-income countries. POCUS presents a practical option, combining diagnostic utility with affordability, safety, and portability. As the healthcare landscape continues to evolve, integrating POCUS into the diagnostic toolkit for paediatric TB could significantly improve early detection and treatment outcomes, particularly in resource-constrained settings. This paper aims to propose a simplified methodology for using mediastinal POCUS in the diagnosis of paediatric TB, and highlight its potential to address current diagnostic challenges and improve care for children affected by TB [44].

## Methods

We present a methodological approach to studying mediastinal lymph nodes for various aetiologies, with a focus on pulmonary tuberculosis (PTB) in children, as it predominantly affects lymph nodes. This approach aims to standardize the technique, improve consistency, mitigate the user-dependent nature of POCUS, and improve the reliability of identifying mediastinal lymphadenopathy.

### Key findings in mediastinal POCUS in paediatric TB

Lymphadenopathy is the most common single manifestation of PTB in children, particularly those under 3 years of age, where its prevalence is highest [45–47]. Studies have indicated that the lymph nodes typically exceed 10 mm in size, larger than those observed in cases of pneumonia [36,48]. Mediastinal lymph node involvement is common in primary PTB in children, occurring in over 80% of cases. The paratracheal, hilar, and subcarinal regions are the most frequent sites of lymphadenopathy in paediatric PTB [18,36,48].

The effectiveness of mediastinal POCUS in diagnosing paediatric TB varies significantly with age. POCUS is particularly useful in infants and young children under 5 years old due to their smaller chest structures, more cartilaginous ribs, and relatively larger thymus. These features allow for clearer imaging of mediastinal lymph nodes, which are often involved in TB. At this age, CXRs may struggle to detect subtle TB-related changes, making POCUS a valuable alternative imaging modality or complement [49–51].

As children grow older, typically around 6 to 10 years and beyond, their chest anatomy becomes more complex—including features of increasing bone density, shrinking thymus, and a developing rib cage. This makes POCUS imaging more challenging, and other diagnostic tools like CXRs or CT scans may be favoured for detailed views of lung structures. Moreover, in older children and adolescents, ultrasound may not penetrate as effectively due to these anatomical changes, reducing its diagnostic accuracy for deeper mediastinal structures [49–51].

Despite these age-related differences, the technique itself remains largely the same, although adjustments may be needed in probe selection and depth settings to ensure optimal image quality. The procedure is relatively quick, typically taking 20–25 minutes if the child cooperates and the operator is experienced. It is a painless, non-invasive technique, though some children may find it uncomfortable or irritating due to the need to remain still, which can extend the duration of the scan.

### Machine specifications

Fixed or portable ultrasound machines with Doppler capabilities (to differentiate blood vessels from lymph nodes) should be used when available. However, Doppler is not strictly necessary because cardiac movements can sometimes produce artifacts. Convex multifrequency or small sectorial cardiac probes are recommended for studying the mediastinum in paediatric patients. High-frequency linear probes are not strictly necessary.

### Depth and focus

In order to increase frame rate, it is recommended to adjust depth to the area of interest, decrease the number of foci, and shrink the lateral field of vision. To establish anatomic references and to determine whether lesions are cystic or solid (echogenicity), the field of study should include a large vessel or cardiac chamber as a landmark.

### Regions covered and consensus classification

**Figure 1.**
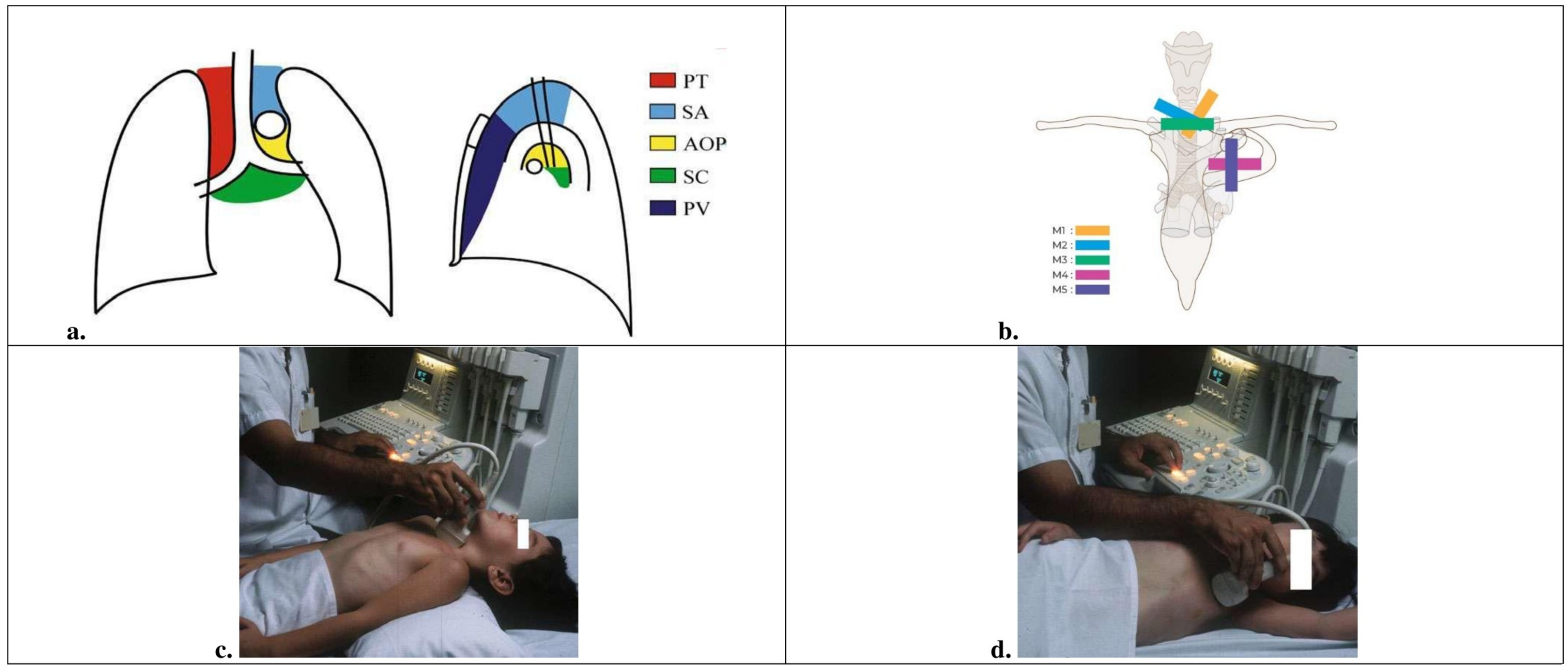
Basics of mediastinal POCUS approach in children. a) Diagram showing POCUS division of the anterior and middle radiologic mediastinal regions: PV, prevascular; PT, paratracheal; SA, supraaortic; AOP, aortopulmonary; SC, subcarinal. The anterior mediastinum—also known as the prevascular (PV) region is in front of the superior vena cava, aorta and pulmonary artery and behind the sternum. The middle mediastinum—further divided into 4 regions: 1) Paratracheal (PT) region—right paratracheal area; 2) Supra-aortic (SA) region—upper portion of the left paratracheal region, above the aortic arch; 3) Aortopulmonary (AOP) region—the area below the aortic arch and above the right pulmonary artery and the left bronchus; 4) Subcarinal (SC) region—behind the bifurcation of the pulmonary artery, above the left atrium in front of the oesophagus and below the carina. The posterior mediastinum—situated in the pre– and paravertebral spaces. From: Pediatric Chest Imaging, edited by Javier Lucaya and Janet L. Strife, © Springer-Verlag Berlin Heidelberg 2008. Reproduced with permission of the Licensor through PLSclear. b) Five standard sonographic slices of the anterior and middle mediastinum: three suprasternal view (M1, M2, M3) and two parasternal views (M4, M5). Prepared by Marie Delorme. c) and d) Patient positioning for POCUS access to the mediastinum using the suprasternal and left parasternal approaches. From: Pediatric Chest Imaging, edited by Javier Lucaya and Janet L. Strife, © Springer-Verlag Berlin Heidelberg 2008. Reproduced with permission of the Licensor through PLSclear.

In order to properly assess the mediastinum, we recommend the following classification of the regions [44]. With the present protocol we aim to visualize the anterior and middle compartments of the mediastinum, divided into the paratracheal region, supra-aortic region, aortopulmonary region, subcarinal region, and posterior mediastinum (Figure 1a).

### Brief description on access windows

A suprasternal approach and a left parasternal approach provide good access to the anterior and middle mediastinum. Supraclavicular, subxiphoid, and subcostal approaches can also be used. A complete sonographic view of the anterior and middle regions of the mediastinum requires five standard slices: three via the suprasternal approach (M1-M3), and two via the left parasternal approach (M4-M5) (Figure 1b). The posterior mediastinum is not ideal for POCUS examination. The diseases found in this region are mostly neurogenic masses and are best examined by CT or medical resonance imaging scan.

### Patient preparation and positioning

When scanning through the suprasternal notch, the child should lie in supine position with a pillow under the shoulders with the head tilted backwards to reach the maximum hyperextension (for M1, M2 and M3). The child should be placed in the left lateral position when scanning through the left parasternal notch (for M4 and M5). It is recommended to place the child in the left lateral position, to displace the mediastinum downwards to increase the acoustic window (Figure 1c and Figure 1d).

## Protocol of the mediastinal POCUS examination approach

### M1 – Oblique suprasternal parasagittal view

**Figure 2.**
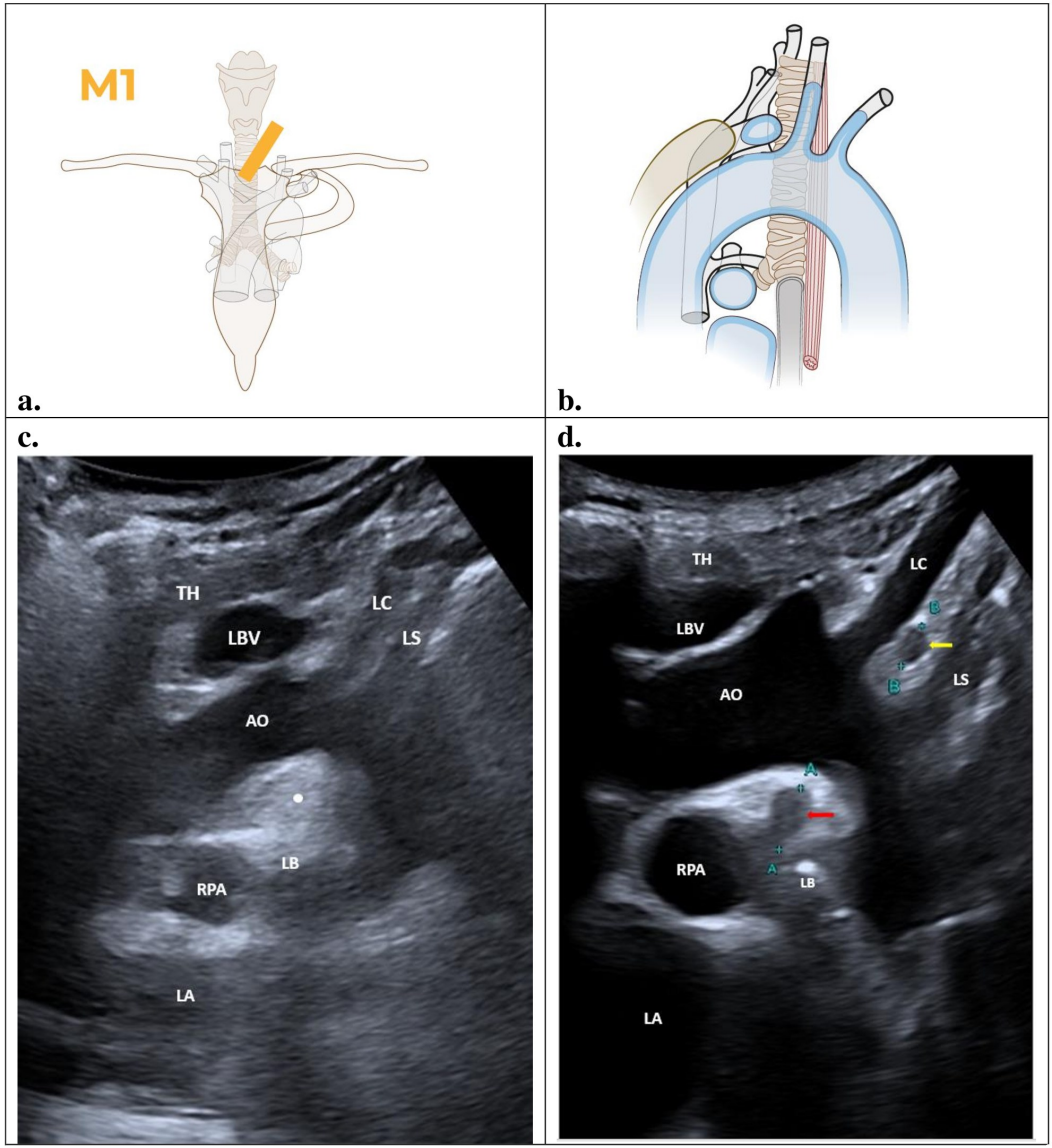
Oblique suprasternal parasagittal view (M1): TH, thymus; LBV, left brachiocephalic vein; LC, left carotid artery; LS, left subclavian artery; AO, aortic arch; LB, left main bronchus; RPA, right pulmonary artery; LA, left atrium. Illustrations prepared by Marie Delorme

The aortopulmonary region and the supraaortic region can be seen in this view. An ultrasound probe is placed between the trachea and left sternocleidomastoid muscle over the sternal manubrium with the reference short orange line facing down (Figure 2a). The aortopulmonary region is characterized by a half-moon-shaped echogenic image because of the presence of mediastinal fat at this level. Vessels shown in blue (Figure 2b) serve as landmarks and anatomical references in this M1 section. This region is anatomically defined by the inferior part of the aortic arch. At the inferior limit, the right pulmonary artery can be seen anteriorly and the artifact of the left main bronchus posteriorly. Additionally, the section displaying the supraaortic region should include the origins of the carotid and subclavian arteries (Figure 2b).

### Findings

Figure 2b illustrates the expected view in the parasagittal section M1 of the suprasternal oblique. A normal POCUS scan of M1 can be seen in image (Figure 2c). This section illustrates the left brachiocephalic vein (LBV) located anteriorly, above the aortic arch (AO) and in front of the left carotid artery (LC) and left subclavian artery (LS). The upper pole of the thymus (TH) emerges from above the left brachiocephalic vein. Inferior to the aortic arch, the hyperechoic fatty tissue can be seen with a semilunar shape of the aortopulmonary region (white dot) with the left main bronchus (LB) and the right pulmonary artery (RPA) inferiorly. The left atrium (LA) is the anechoic structure inferior to the right pulmonary artery. In some instances, there may be mirror artifact of the left subclavian artery, secondary to the pleural surface of the left pulmonary upper lobe, sometimes seen as a “vascular duplication.” Typically, these mirror artifacts appear as duplicate images of vessels (in this case, the left subclavian artery) or other structures adjacent to the pleura.

Figure 2d is an example of an abnormal scan in the context of PTB: the presence of an oval shape hypoechoic structure just above the left bronchus in the aortopulmonary region (red arrow) and another one between the left carotid artery and the left subclavian artery (yellow arrow), indicate the existence of a lymph node in the aortopulmonary region and the supraaortic region. A lymph node with the size above 1 cm (10 mm) should be considered pathological.

### M2 – Oblique coronal view

**Figure 3.**
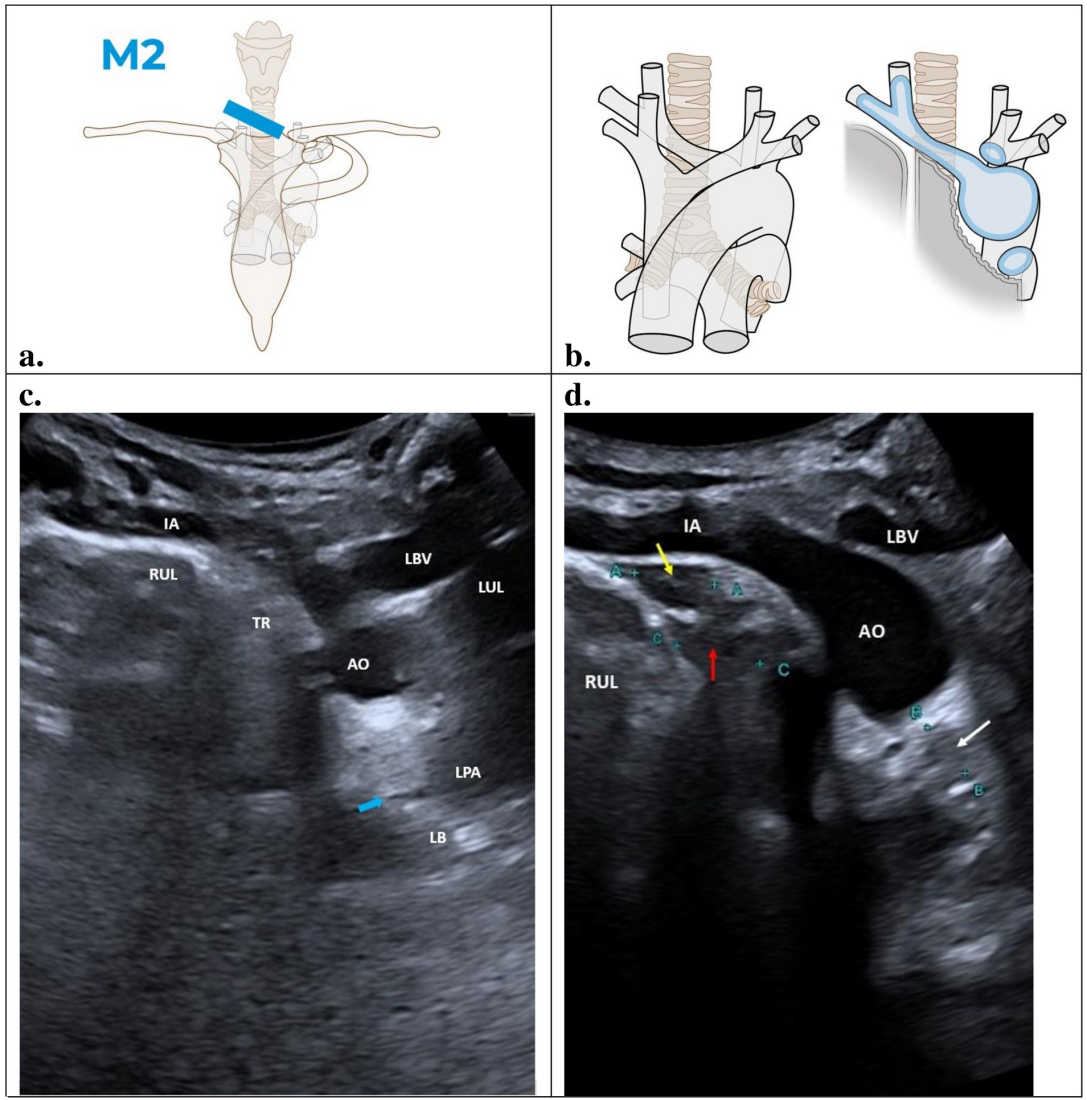
Oblique coronal view (M2): IA, innominate artery; RUL, right upper lobe; TR, trachea; AO, aortic arch; LBV, left brachiocephalic vein; LUL, left upper love; LPA, left pulmonary artery; LB, left bronchus. Illustrations prepared by Marie Delorme.

This section shows the paratracheal region between the upper right pulmonary lobe, the trachea, and the aortopulmonary region below the aorta. The probe should be placed in the suprasternal notch, on the right of the trachea, and angled downwards toward the mediastinum (Figure 3a). A slight compression can be applied on the right sternocleidomastoid muscle. Anatomically, this region is defined by the vascular landmark of the innominate artery, the right upper lobe pleural surface, the trachea, and the upper margin of left bronchus. These anatomical features should be included in this view, as depicted in blue in Figure 3b. The paratracheal region is a virtual space formed by the upper lobe's pleural surface that contacts with the right wall of the trachea.

### Findings

Figure 3b shows the key structural features of M2 section. A normal POCUS image of the suprasternal, oblique coronal view (M2) is shown in Figure 3c. An echogenic convex line from the pleural surface of the right upper lung lobe (RUL) can be seen on the right side of the scan. The innominate artery (IA) branches from the aortic arch and divides into the right common carotid and right subclavian arteries above the pleural line of the right upper lung lobe. A hypoechogenic imaging representing the shadowing artifact produced by the trachea (TR) lies at the end of the pleural line between the right upper lung lobe and the aorta. Additionally, the echogenic stepladder structures can be observed extending downwards from the trachea (blue arrow). These are the tracheo-bronchial cartilages which terminate below the aortopulmonary region. Tracheal and bronchial cartilage produces parallel echogenic stepladders, also known as “stair artifacts.”

The left brachiocephalic vein (LBV) is an anechoic structure above the left upper margin of the aorta. The anechoic left pulmonary artery (LPA) is located to the left of the aortopulmonary region. The artifact of the left upper lung lobe (LUL) is located to the left of the left brachiocephalic vein and left pulmonary artery. Aortopulmonary region is formed in this M2 section by an echogenic fatty triangle between the aorta, trachea-left bronchus (LB), and left pulmonary artery.

When lymph nodes appear in the paratracheal region there is a separation between the convex echogenic line from the right upper lobe and the hypoechoic artifact of the trachea. This convex echogenic line becomes a concave line. Lymph nodes are hypoechoic and sometimes confluent (yellow and red arrows) (Figure 3d). Lymph nodes can also be seen in the aortopulmonary region, which has a triangular shape in this M2 section above the left tracheobronchial wall (white arrow) (Figure 3d).

### M3 – Coronal view

**Figure 4.**
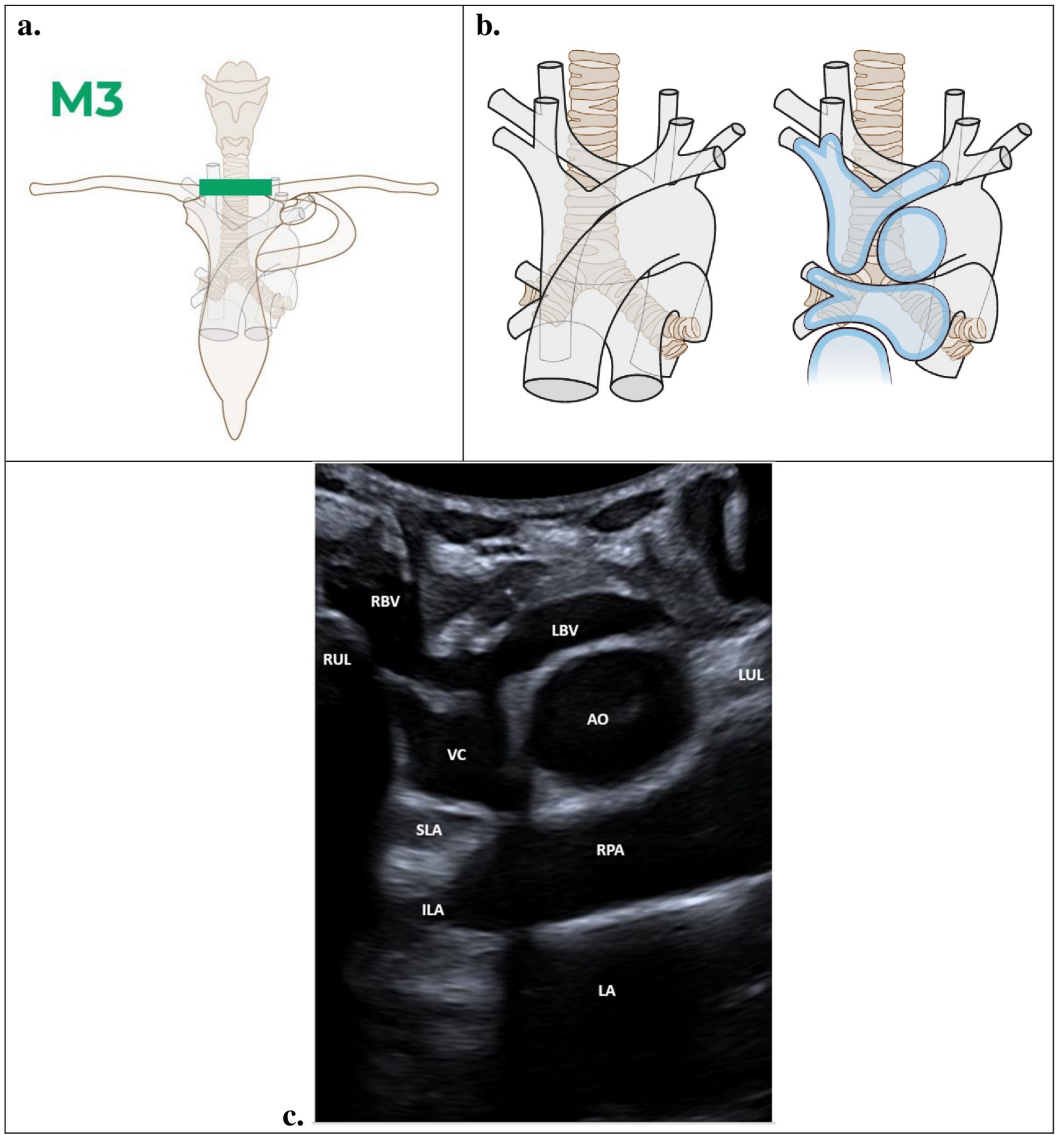
Coronal view (M3): RBV, right brachiocephalic vein; LBV, left brachiocephalic vein; RUL, right upper lobe; VC, vena cava; AO, aortic arch; LUL, left upper lobe; SLA, superior lobe artery; ILA, inferior lobe artery; RPA, right pulmonary artery; LA, left atrium. Illustration prepared by Marie Delorme.

The M3 section does not traverse any nodal territories and is composed entirely of blood vessels. This view is used for visualization of vessels but not for studying a specific mediastinal region. It is a good section to see pericardial recesses that may enlarge when tuberculous lymph nodes appear in the mediastinum. The probe is placed in the central suprasternal region in a completely coronal section (Figure 4a). In this view, the right pulmonary artery and its bifurcation, superior vena cava, aorta and left atrium, depicted in blue (Figure 4b). The M3 section is significant because it allows for confirmation of the most anterior coronal anatomical limit via a suprasternal approach, serving as an indicator of good suprasternal percutaneous access with a good acoustic window.

### Findings

An illustration of the suprasternal coronal view (M3) is shown in Figure 4b. Figure 4c shows a normal POCUS image of the M3 view. In the center, the anechoic v-shaped vena cava (VC) with right brachiocephalic vein (RBV) branches to the right and left brachiocephalic vein (LBV) branches to the left. The superior pole of thymus arises above the venous trunks. The aorta (AO) is located on the left lateral of the vena cava and inferior to the left brachiocephalic vein. The anechoic right pulmonary artery (RPA) and its bifurcations—the superior lobe artery (SLA) and inferior lobe artery (ILA)—occur inferiorly and posteriorly to the vena cava and aorta. Below the right pulmonary artery is the anechoic left atrium (LA). Located below the left brachiocephalic vein, the pleural artifact of the left upper lung lobe (LUL) abuts the left margin of the aorta. Immediately inferior to the right brachiocephalic vein there is the pleural artifact of the homogeneous right upper lung lobe (RUL).

A small triangular region corresponding to fat around the right pulmonary hilum may lie between the upper and lower lobes of the right pulmonary artery. Exceptionally, when this region is visible and the normal hyperechoic triangle is absent, the presence of lymph nodes cannot be excluded.

### M4 – Axial view

**Figure 5.**
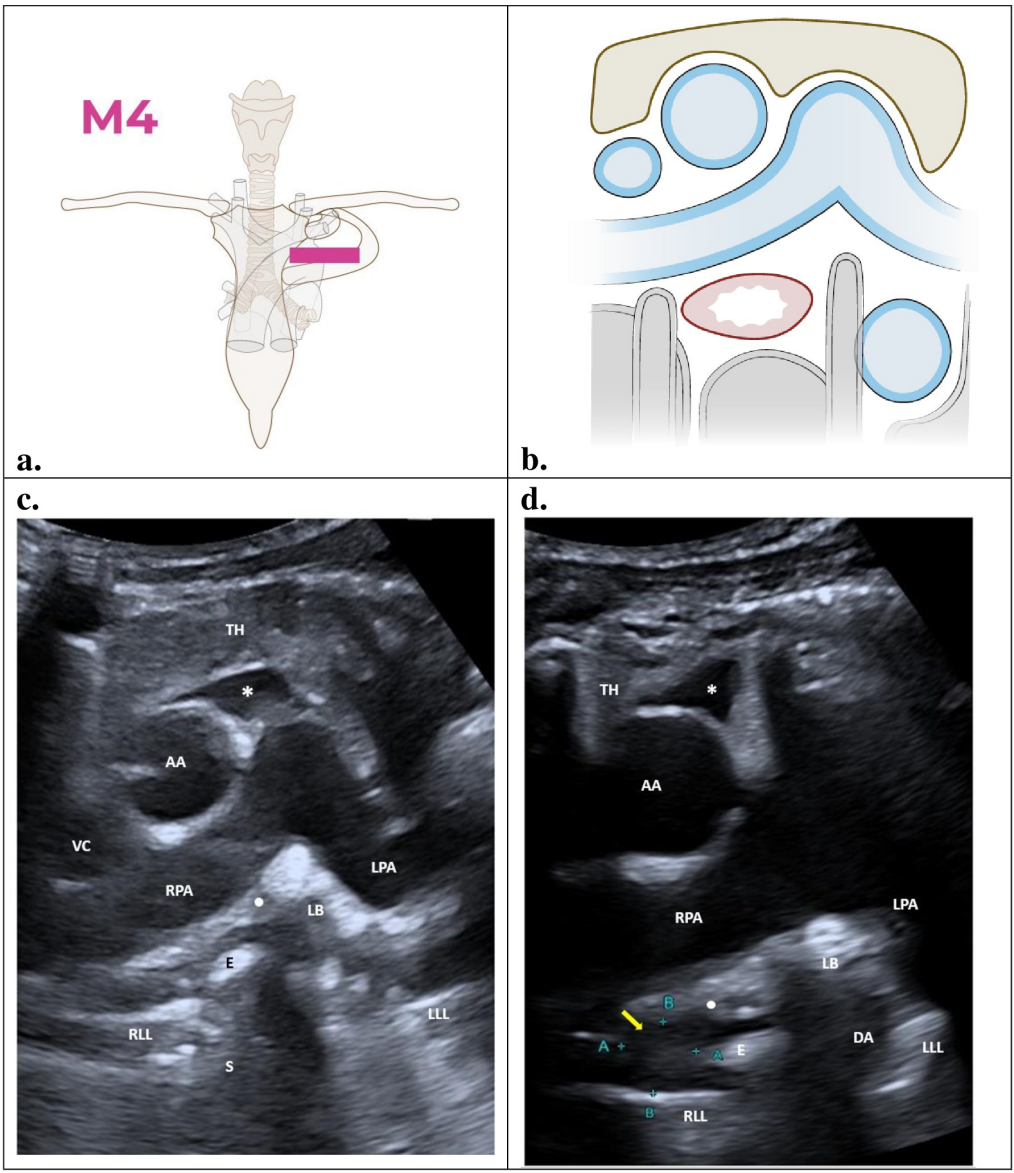
Axial view (M4): TH, thymus; AA, ascending aorta; VC, vena cava; RPA, right pulmonary artery; LPA, left pulmonary artery; LB. left main bronchus; E, oesophagus; RLL, right lower lobe; LLL, left lower lobe; S, spine; DA, descending aorta. Illustrations prepared by Marie Delorme.

This sonographic section is similar to CT axial slices obtained in this area, allowing study of the subcarinal and prevascular regions. During the examination, the probe is positioned in the second intercostal space with the reference bar towards the left margin of the sternum (Figure 5a). In newborns, the transsternal approach can be used because their sternum has not yet fully calcified. This view can be interpreted using the pulmonary artery bifurcation as the anatomical reference (Figure 5b).

### Findings

Figure 5b illustrates in blue which key structures should be visualized in the left parasternal axial view (M4). In the normal POCUS image of M4 view, the thymus (TH) on the superior aspect of the image (Figure 5c) is shown with a homogeneous echotexture. The presence of the thymus improves and expands the acoustic window for accessing the mediastinum. The thymus gradually decreases in size with age. Anatomically, the thymus corresponds to the prevascular region of the anterior mediastinum, which in young children is completely occupied by this organ.

The ascending aorta (AA) and the superior pericardial recess (*), located posterior to the thymus, appear as a rounded and triangular anechoic structures (respectively). The anechoic vena cava (VC) lies on the right of the ascending aorta. Posterior to the thymus and to the left of the ascending aorta is the bifurcation of the main pulmonary artery into the right (RPA) and left (LPA) pulmonary arteries. The oesophagus (E) has a thick hypoechogenic concentric wall and is found below the right pulmonary artery in the subcarinal space. The normal oesophagus must not be misinterpreted as a subcarinal solid mass. It can be identified by asking the patient to swallow saliva or fluid during the examination. An echogenic image of saliva can then be seen tracking down, excluding a mass in the mediastinum. It is possible to visualize the passage of gastric content from the stomach to the oesophagus in patients with gastroesophageal reflux in real time.

The pleural artifact of the right lower lung (RLL) is on the right lower edge of the oesophagus, and the bone artifact of the spine (S) posteriorly. The descending aorta (DA) appears as a rounded, anechoic structure, but clear visualization is challenging due to shadowing artifact of the left main bronchus (LB). The pleural artifact of the left lower lung (LLL) lies to the left of this.

The image (Figure 5d) shows a hypoechoic lymph node on the right of the oesophagus (yellow arrow). A superior pericardial recess (*) can be seen on the image, mimicking lymphadenopathy. Usually triangular in shape, the recess is anechoic, and the size changes with the heartbeat, allowing it to be differentiated from lymphadenopathy. The white dot (Figure 5c and Figure 5d) indicates the subcarinal region between the right pulmonary artery and the oesophagus. In Figure 5d, the lymph node is located in the subcarinal region, to the right of the oesophagus.

### M5 – Parasagittal view through the left parasternal approach

**Figure 6.**
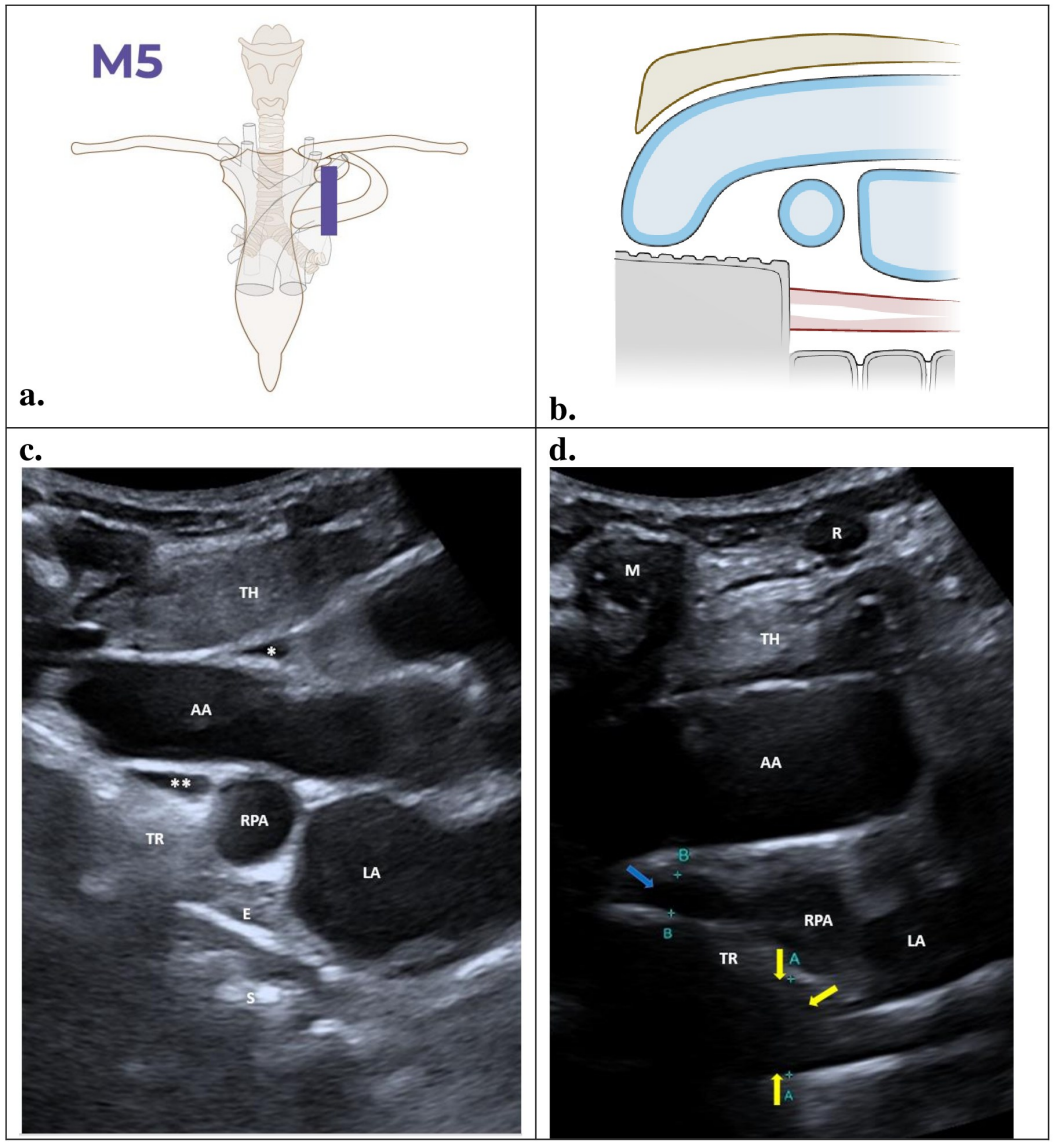
Parasagital view (M5): TH, thymus; AA, ascending aorta; TR, trachea; RPA, right pulmonary artery; LA, left atrium; E, oesophagus; S, spine; M, sternal manubrium; R, cartilaginous part of the parasternal rib. Illustrations prepared by Marie Delorme.

This M5 view is used to study the subcarinal and prevascular regions. The probe should be positioned alongside the sternum in the second to third intercostal spaces with the reference bar facing the head (Figure 6a). The anatomic references used are the ascending aorta (depicted in blue), the trachea, and the oesophagus (Figure 6b). Making the patient swallow saliva, milk or water can help identify the oesophagus.

### Findings

The scan image (Figure 6c) shows a normal left parasternal parasagittal view. The homogenous thymus (TH) can be seen in the superior aspect of the image. The anechoic ascending aorta (AA) lies posterior to the thymus. The anechoic right pulmonary artery (RPA) is central, rounded in shape, located posterior to the ascending aorta, and superior to the anechoic left atrium (LA). The hyperechoic artefact of the trachea lies posterior to the ascending aorta, and the hypoechogenic oesophagus (E) arises from behind the artifact of the trachea. It has an echogenic artifact generated by saliva. Posterior is the irregular artifact from the spine (S). In this section, the pericardial recesses can be seen, along with the anterior recess of the pericardium (*) and the retro aortic portion of the transverse recess of the pericardium (**).

An M5 abnormal scan (Figure 6d) shows hypoechoic lymph nodes in the subcarinal region, down to the artifact of the trachea (yellow arrow). Additionally, in this section, lymph nodes can be observed in the pre-tracheal region, covering the anterior wall of the trachea (blue arrow). The lymph nodes located anterior to the trachea provide contiguity between the aortopulmonary window and the subcarinal space, positioned in front of the carina.

Anterior to the thymus on the left is the anechoic cartilaginous portion of the parasternal rib (R), which, due to its lack of calcification, creates an acoustic window ideal for performing a parasternal approach in mediastinal POCUS. On the right, the shadow of the sternal manubrium (M) is visible, caused by the calcification of the manubrium. (Supplementary Material - Table S1)

### Technical considerations

For lung POCUS, the linear probe is suitable. For mediastinal POCUS, convex or micro-convex probes are suitable for the suprasternal or parasternal approaches. In early newborns, a linear probe is useful for the parasternal approach [38,44,52]. A colour Doppler ultrasound machine is recommended for distinguishing blood vessels from masses like lymph nodes. However, in the mediastinum, the effectiveness of this technology can be limited due to interference from cardiac movement. Blood flow in a vessel can be observed using colour Doppler, which cannot be seen in masses or recesses [44].

On mediastinal POCUS, TB-specific lymph node findings may include the target sign, which is characterized by hypoechoic necrotic centres surrounded by hyperechoic tissue and is indicative of active disease. Chronic TB lymph nodes often present as calcified nodes, and appear as hyperechoic structures with acoustic shadowing [53,54]. These features aid in distinguishing TB-related lymphadenopathy from other causes.

The proposed POCUS methodology for paediatric PTB diagnosis offers significant potential but has limitations. Additionally, mediastinal POCUS on extremely malnourished children presents several challenges. The disappearance of fatty connective tissue reduces the acoustic window, making it harder to obtain clear images. While the presence of lymph nodes can expand the acoustic window by becoming more visible and allowing better ultrasound transmission, their absence means the lungs occupy the space, closing the acoustic window. The smaller anatomical structures in children can also limit the resolution and signal available for imaging. Furthermore, malnourished children may be more irritable and less cooperative during the procedure, which can complicate the imaging process [55–57].

In a previous paper, Pool et al. discussed the technical aspects of mediastinal ultrasound [38]. Considering that zones A-H do not correspond to any nodal or mediastinal regions, the proposed division of sections by these authors is—in our opinion—not practical for POCUS users. We do not support the conclusion that there can be no visualisation of the right mediastinum using the mediastinal approach. Furthermore, no mention is made of pericardial recesses in the article. An easy-to-follow, practical approach to mediastinal POCUS is presented in this paper for the diagnosis of childhood PTB. A significant feature of this method is that it is based on anatomical mediastinal regions.

## Future perspective

Future research should focus on refining POCUS techniques, integrating them into existing TB diagnostic frameworks, and evaluating their impact on clinical outcomes. Standardized training and certification programs are crucial to ensure quality and consistency, especially in low- and middle-income countries. Expert-guided training and access to proper equipment are essential for successful implementation. In the future, virtual simulators could be useful for training purposes. Additionally, pooling data for machine learning and artificial intelligence analyses will make it easier for non-experts to use, reduce diagnostic time, and improve decision-making.

## Conclusion

Mediastinal POCUS is invaluable for studying lymph node disease, particularly in paediatric TB. Given the challenges in diagnosing paediatric TB, this approach enables accurate diagnosis and monitoring of treatment response. POCUS is clinician-performed, cost-effective, non-invasive, portable, and non-ionizing; making it ideal for TB control programs in resource-limited settings.
