# Supplementary material for: POCUS Examination of the Mediastinum in Children: A Simplified and Standardized Protocol for Pulmonary Tuberculosis
Source: POCUS J. 2025 Nov 17;10(2):123–34. doi: 10.24908/pocusj.v10i02.19176 (PMC12658583; doi:10.24908/pocusj.v10i02.19176)
Supplement: Supplementary file 1 [file pocusj-10-02-19176-s001.pdf]

Supplementary table 1: Mediastinal ultrasound approach and findings

Aspect	Suprasternal notch	Left parasternal notch
Patient Preparation and Positioning	Child in supine position with pillow under shoulders, head tilted back for M1, M2, and M3	Child in left lateral position for M4 and M5. Left lateral position recommended to displace mediastinum downwards to the left and increase acoustic window.
**Step**	**Position**	**Findings**
**M1 View: Oblique Parasagittal**	Probe between trachea and left sternocleidomastoid muscle over the sternal manubrium.	Supraaortic and aortopulmonary regions. Aortopulmonary region seen as a half-moon shaped echogenic fatty tissue.<br>Normal scan shows LBV, AO, LC, LS, RPA, LA and LB. Mirror artifacts.<br><br>Abnormal scan shows lymph node (LN).
**M2 View: Oblique Coronal**	Probe between trachea and right sternocleidomastoid muscle over the sternal manubrium.	Paratracheal and aortopulmonary regions.<br>IA, RUL, trachea, LB, LPA, LBV, LUL. Step artifacts from left tracheobronchial wall.<br><br>Pathological scan shows pleural line distortion from convex to concave, that indicates lymphadenopathy.
**M3 View: Coronal**	Probe in central suprasternal region, coronal section.	No ganglionar regions. Right hilum. V-shaped SVC with RBV and LBV, AO, RPA and bifurcations, LUL, RUL. Pericardial recesses.
**M4 View: Axial**	Probe in second intercostal space, reference dot towards sternum.	Prevascular and subcarinal regions. TH, AA, VC, RPA, LPA, E, RLL, spine, DA, LLL. Oesophagus is identified by swallowing.<br><br>Abnormal scan shows subcarinal lymph node.
**M5 View: Parasagittal**	Probe alongside sternum in 2nd–3rd intercostal spaces, reference dot towards head.	Prevascular and subcarinal regions. TH, AA, RPA, LA, TR, E. Oesophagus is identified by swallowing.<br><br>Abnormal scan shows lymph node in the subcarinal region.
